# NAMPT-dependent NAD^+^ salvage is crucial for the decision between apoptotic and necrotic cell death under oxidative stress

**DOI:** 10.1038/s41420-022-01007-3

**Published:** 2022-04-11

**Authors:** Takuto Nishida, Isao Naguro, Hidenori Ichijo

**Affiliations:** Laboratory of Cell Signaling, Graduate School of Pharmaceutical Science, The University of Tokyo, 7-3-1 Hongo, Bunkyo-ku, Tokyo, Japan

**Keywords:** Cell death, Stress signalling, DNA damage response

## Abstract

Oxidative stress is a state in which the accumulation of reactive oxygen species exceeds the capacity of cellular antioxidant systems. Both apoptosis and necrosis are observed under oxidative stress, and we have reported that these two forms of cell death are induced in H_2_O_2_-stimulated HeLa cells depending on the concentration of H_2_O_2_. Weak H_2_O_2_ stimulation induces apoptosis, while strong H_2_O_2_ stimulation induces necrosis. However, the detailed mechanisms controlling the switching between these forms of cell death depending on the level of oxidative stress remain elusive. Here, we found that NAD^+^ metabolism is a key factor in determining the form of cell death in H_2_O_2_-stimulated HeLa cells. Under both weak and strong H_2_O_2_ stimulation, intracellular nicotinamide adenine dinucleotide (NAD^+^) was depleted to a similar extent by poly (ADP-ribose) (PAR) polymerase 1 (PARP1)-dependent consumption. However, the intracellular NAD^+^ concentration recovered under weak H_2_O_2_ stimulation but not under strong H_2_O_2_ stimulation. NAD^+^ recovery was mediated by nicotinamide (NAM) phosphoribosyltransferase (NAMPT)-dependent synthesis via the NAD^+^ salvage pathway, which was suggested to be impaired only under strong H_2_O_2_ stimulation. Furthermore, downstream of NAD^+^, the dynamics of the intracellular ATP concentration paralleled those of NAD^+^, and ATP-dependent caspase-9 activation via apoptosome formation was thus impaired under strong H_2_O_2_ stimulation. Collectively, these findings suggest that NAD^+^ dynamics balanced by PARP1-dependent consumption and NAMPT-dependent production are important to determine the form of cell death activated under oxidative stress.

## Introduction

Apoptosis and necrosis are the most typical classifications of the forms of cell death, and a prominent difference between these forms of cell death is plasma membrane integrity [1]. In general, apoptosis is not accompanied by plasma membrane collapse, and apoptotic cells are actively removed by phagocytosis in the organism [2, 3]. On the other hand, necrosis is accompanied by plasma membrane disruption, resulting in leakage of intracellular molecules such as damage-associated molecular patterns (DAMPs). Since DAMPs are potent activators of inflammatory responses, necrosis is a more proinflammatory form of cell death than apoptosis [4]. Although normal inflammation is critical for biological defense systems, excess inflammation often causes disease [5, 6]. For example, pyroptosis, a type of regulated necrosis induced by bacterial infection, activates the immune system to remove pathogens via the release of intracellular molecules [7–10]. On the other hand, excessive necrosis leads to pathogenic inflammation, resulting in exacerbation of certain disorders, such as liver disease [11–13]. Therefore, proper selection of the cell death form is critical for maintaining an organism’s homeostasis.

The mechanisms underlying the switching of cell death between apoptosis and necrosis have been reported in several contexts. Necroptosis, another type of regulated necrosis, is induced as an alternative to apoptosis when caspase-8 is inhibited under TNFα stimulation [14, 15]. It has also been reported that the preexisting intracellular ATP concentration is critical in determining the form of cell death induced by the Fas ligand and hypoxia [16–18]. These studies showed that intracellular ATP is required for the induction of apoptosis and that necrosis is induced when the intracellular ATP supply is exhausted. This switch in the form of cell death might manifest as the appearance of necrotic cells mixed with apoptotic cells in areas with a decreased concentration of ATP in vivo, such as the center of solid tumors and ischemic tissues.

We have previously reported that weak oxidative stress induces apoptosis, whereas strong oxidative stress induces necrosis in HeLa cells. Strong oxidative stress-induced necrosis was mediated by the ASK1-p38-NR4A2 signal transduction [19]. However, the detailed mechanisms controlling the switch between the form of cell death depending on the strength of oxidative stress remain elusive. Here, we investigated the forms of cell death induced under various levels of oxidative stress and found that the NAMPT-dependent NAD^+^ metabolism under oxidative stress is a crucial factor determining the form of cell death. We also found that the dynamics of the intracellular ATP concentration paralleled those of NAD^+^, which is important for triggering the intrinsic apoptotic pathway through cleavage of caspase-9 and -3 under weak oxidative stress.

## Results

### Intracellular ATP recovers only under weak H_2_O_2_ stimulation

**Fig. 1:**
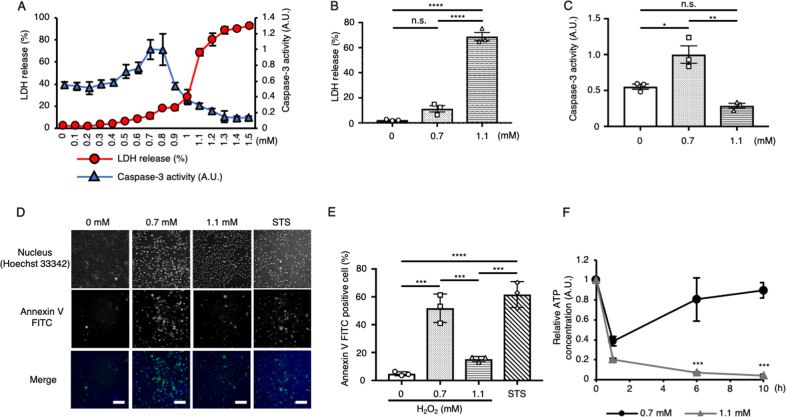
The intracellular ATP concentration is recovered only under weak H_2_O_2_ stimulation. **A** LDH release into the conditioned medium and caspase-3 activity of HeLa cells stimulated with various concentrations of H_2_O_2_ for 30 h and 10 h, respectively (*n* = 3). **B** LDH release after 0, 0.7 and 1.1 mM H_2_O_2_ stimulation (data extracted from panel **A**). **C** Caspase-3 activity after 0, 0.7 and 1.1 mM H_2_O_2_ stimulation (data extracted from panel **A**). **D** Representative images of Annexin V-stained HeLa cells stimulated with H_2_O_2_ or STS for 20 h. Blue; Hoechst 33342, green; Annexin V FITC. Scale bar, 100 μm. **E** Percentage of Annexin V-positive cells, as detected by image analysis (*n* = 3). **F** The dynamics of the relative intracellular ATP concentration in HeLa cells stimulated with 0.7 mM or 1.1 mM H_2_O_2_ (n = 3). **B**, **C**, **E** *P < 0.05, **P < 0.01, ***P < 0.001 and ****P < 0.0001 by one-way ANOVA followed by the Tukey-Kramer multiple comparisons test. **F** ***P < 0.001 by two-way ANOVA followed by the Sidak correction for multiple comparisons compared with 0.7 mM H_2_O_2_ stimulation. All data are presented as the mean ± SEM values.

We reported in a previous study that the form of cell death can be switched from apoptosis to necrosis by increasing the concentration of H_2_O_2_ in HeLa cells [19]. To estimate the threshold of H_2_O_2_ concentration for the switch from apoptosis to necrosis, we examined the effects of various concentrations of H_2_O_2_ and detected necrosis and apoptosis by measuring LDH release (30 h after stimulation) and caspase-3 activity (10 h after stimulation), respectively (Fig. 1A). LDH release was simply increased in an H_2_O_2_ concentration-dependent manner, and the percentage of LDH release in response to stimulation with 1.1 mM H_2_O_2_ was approximately 70% (Fig. 1A, B). On the other hand, caspase-3 activity peaked in response to stimulation with 0.7–0.8 mM H_2_O_2_, and the percentage of LDH release was less than 20% under this treatment (Fig. 1A). Caspase-3 activity started to decrease at 0.9 mM H_2_O_2_ and further decreased along with concentrations over 0.9 mM, becoming even lower than that in the basal state (Fig. 1A, C). To evaluate the induction of apoptosis by another method, HeLa cells were stained with Annexin V FITC at 20 h after exposure to 0.7 or 1.1 mM H_2_O_2_ [20]. Staurosporine (STS) treatment was used as a positive control for apoptosis induction. The ratio of Annexin V FITC-positive cells was increased in cells treated with 0.7 mM H_2_O_2_ to an extent comparable to that observed in STS-treated cells. On the other hand, consistent with caspase-3 activity, the number of Annexin V FITC-positive cells was low after 1.1 mM H_2_O_2_ stimulation (Fig. 1D, E). Collectively, these results suggest that 0.7 mM H_2_O_2_ stimulation mainly induces apoptosis, while 1.1 mM H_2_O_2_ stimulation mainly induces necrosis in HeLa cells.

Previous studies have suggested that the form of cell death in response to certain stimuli is affected by the intracellular ATP concentration; apoptosis is induced when the preexisting intracellular ATP concentration is high, whereas the same stimuli induce necrosis when the intracellular ATP concentration is low [16, 18]. Therefore, we examined the concentration of intracellular ATP at various time points under 0.7 mM and 1.1 mM H_2_O_2_ stimulation (Fig. 1F). Under both 0.7 and 1.1 mM H_2_O_2_ stimulation, the ATP concentration sharply decreased immediately after stimulation (approximately 1 h after stimulation: early phase). However, the ATP concentration gradually recovered under 0.7 mM stimulation but not under 1.1 mM stimulation. These results suggest that the ATP concentration at the late phase (after 6 h) of stimulation may be a key determinant of the form of cell death under H_2_O_2_ stimulation.

### Intercellular NAD^+^ is consumed by PARP1 in both weak and strong H_2_O_2_ stimulation, but it recovers only in the late phase of weak stimulation

**Fig. 2:**
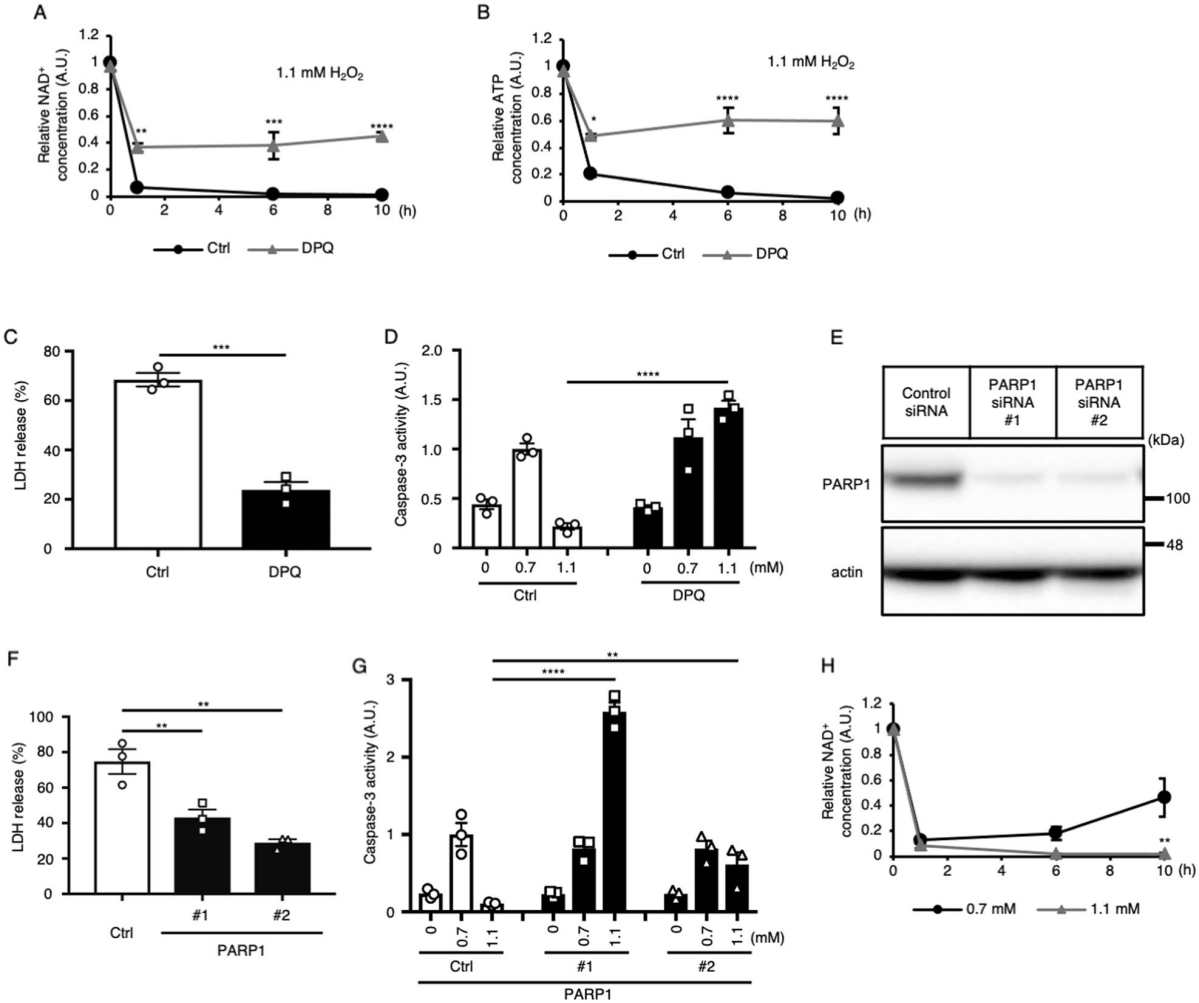
NAD^+^ amount in the late phase of H_2_O_2_ stimulation determines the form of cell death. **A**, **B** Effect of a PARP1 inhibitor (DPQ) on the dynamics of the relative intracellular concentrations of NAD^+^ **A** and ATP **B** in HeLa cells stimulated with 1.1 mM H_2_O_2_ (*n* = 3). **C** Effect of DPQ on LDH release in HeLa cells stimulated with 1.1 mM H_2_O_2_ (*n* = 3). **D** Effects of DPQ on caspase-3 activity in HeLa cells stimulated with 0.7 mM or 1.1 mM H_2_O_2_ (*n* = 3). **E** Knockdown efficiency of PARP1 by siRNA, as determined by immunoblotting (*n* = 3). **F** Effect of PARP1 knockdown on LDH release in HeLa cells stimulated with 1.1 mM H_2_O_2_ (*n* = 3). **G** Effects of PARP1 knockdown on caspase-3 activity in HeLa cells stimulated with 0.7 mM or 1.1 mM H_2_O_2_ (*n* = 3). **H** The dynamics of the relative intracellular NAD^+^ concentration in HeLa cells stimulated with 0.7 mM or 1.1 mM H_2_O_2_ (*n* = 3). **A**, **B**, **D**, and **H** *P < 0.05, **P < 0.01, ***P < 0.001 and ****P < 0.0001 by two-way ANOVA followed by the Sidak correction for multiple comparisons compared with the control. **C** ***P < 0.001 by unpaired two-tailed Student’s *t*-test compared with the control. **F** **P < 0.01 by one-way ANOVA followed by Dunnett’s multiple comparisons test compared with the control. **G** **P < 0.01 and ****P < 0.0001 by two-way ANOVA followed by Dunnett’s multiple comparisons test compared with the control. All data are presented as the mean ± SEM values.

Previous reports have shown that DNA-damaging stimuli decrease the intracellular ATP concentration through poly (ADP-ribose) (PAR) polymerase 1 (PARP1) activation [21, 22]. PARP1 synthesizes PAR from NAD^+^ [23]. Although PARP1 activity is essential for DNA repair, overactivation of PARP1 by excessive DNA damage causes depletion of NAD^+^ [24]. Since NAD^+^ is an important coenzyme in ATP generation, NAD^+^ depletion results in a low-energy state and necrotic cell death [21, 22, 25]. PARP1-dependent necrotic cell death is called parthanatos [26]. It has been reported that PARP1 is also activated by oxidative stress [27, 28]. Thus, we examined whether the ATP decline under H_2_O_2_ stimulation is related to NAD^+^ consumption by PARP1 (Fig. 2A, B). Treatment with a PARP1 inhibitor, DPQ, partially prevented the acute decreases in NAD^+^ and ATP at 1 h under 1.1 mM H_2_O_2_ stimulation. No further decrease was observed thereafter, and the state was kept up to 10 h after stimulation (Fig. 2A, B), indicating that PARP1 is required for NAD^+^ and ATP exhaustion under H_2_O_2_ stimulation. As it was suggested that the intracellular ATP concentration in the late phase of stimulation may determine the form of cell death under H_2_O_2_ stimulation (Fig. 1F), we evaluated the effect of PARP1 inhibition on the form of cell death under 1.1 mM H_2_O_2_ stimulation. Consistent with the dynamics of ATP concentration under oxidative stress revealed by us and others [27], PARP1 inhibition not only suppressed LDH release but also increased caspase-3 activity upon stimulation with 1.1 mM H_2_O_2_ (Fig. 2C, D). Moreover, PARP1 depletion by siRNA resulted in the suppression of LDH release and the recovery of caspase-3 activity (Fig. 2E–G). These results suggest that NAD^+^ consumption by PARP1 affects both early and late ATP concentration up to 10 h and thus regulates the decision between apoptotic and necrotic cell death under H_2_O_2_ stimulation. It has been reported that not only depletion of NAD^+^ but also translocation of apoptosis-inducing factor (AIF) from mitochondria to the nucleus is mediated by activation of PARP1 to execute parthanatos [26, 29–33]. However, AIF knockdown did not affect necrosis induction by 1.1 mM H_2_O_2_ stimulation (Supplementary Fig. S1A and B), suggesting that AIF may not be involved in the necrosis induced by 1.1 mM H_2_O_2_ stimulation.

Since the dynamics of ATP in the late phase of H_2_O_2_ stimulation differed depending on the concentration of H_2_O_2_, we compared the dynamics of NAD^+^ under weak and strong H_2_O_2_ stimulation (Fig. 2H). Similar to the ATP dynamics, both 0.7 and 1.1 mM H_2_O_2_ decreased the NAD^+^ concentration in the early phase of the stimulation, whereas the NAD^+^ concentration recovered only in the late phase of 0.7 mM stimulation. These results suggest that the dynamics of intracellular NAD^+^ is the dominant determinant of the dynamics of intracellular ATP at least under H_2_O_2_ stimulation, and that the NAD^+^ concentration in late phase but not that in early phase determines the form of cell death.

### The NAMPT-dependent NAD^+^ salvage pathway is necessary for the recovery of NAD^+^ and induction of apoptosis under weak H_2_O_2_ stimulation

**Fig. 3:**
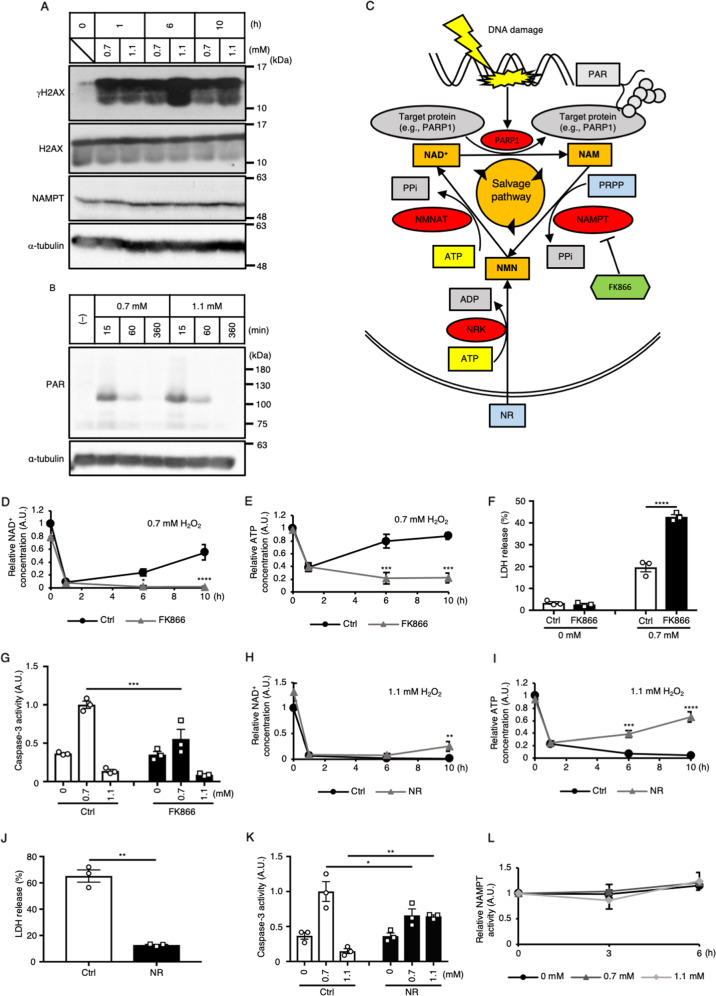
The NAMPT-dependent NAD^+^ salvage pathway is necessary for the recovery of NAD^+^ and induction of apoptosis under weak H_2_O_2_ stimulation. **A** The amounts of endogenous γH2AX and NAMPT proteins under 0.7 and 1.1 mM H_2_O_2_ stimulation were determined by immunoblotting (*n* = 3). **B** Amount of PARylated proteins in H_2_O_2_-stimulated HeLa cells, as determined by immunoblotting (*n* = 3). **C** Schematic drawing of the NAD^+^ salvage pathway in DNA damage. Via DNA damage, PARP1 PARylates target proteins, consuming NAD^+^, and nicotinamide (NAM) is generated as a byproduct. NAMPT converts NAM to nicotinamide mononucleotide (NMN) using 5′-phosphoribosyl-1-pyrophosphate (PRPP). Finally, NAD^+^ is resynthesized from NMN and ATP by NMNAT. Nicotinamide riboside (NR) enters the cell and is converted to NMN by NRK using ATP. **D**, **E** Effect of a NAMPT inhibitor (FK866) on the dynamics of the relative intracellular concentrations of NAD^+^ **D** and ATP **E** in HeLa cells stimulated with 0.7 mM H_2_O_2_ (*n* = 3). **F** Effect of FK866 on LDH release in HeLa cells stimulated with 0.7 mM H_2_O_2_ (*n* = 3). **G** Effect of FK866 on caspase-3 activity in HeLa cells stimulated with 0.7 mM or 1.1 mM H_2_O_2_ (*n* = 3). **H**, **I** Effects of NR treatment on the dynamics of the relative intracellular concentrations of NAD^+^ **H** and ATP **I** in HeLa cells stimulated with 1.1 mM H_2_O_2_ (*n* = 3). **J** Effect of NR treatment on LDH release in HeLa cells stimulated with 1.1 mM H_2_O_2_ (*n* = 3). **K** Effects of NR treatment on caspase-3 activity in HeLa cells stimulated with 0.7 mM or 1.1 mM H_2_O_2_ (*n* = 3). **L** In vitro activity of NAMPT purified from HeLa cells stably expressing Flag-NAMPT under 0, 0.7, or 1.1 mM H_2_O_2_ stimulation (*n* = 3). **D**–**I** and **K** *P < 0.05, **P < 0.01, ***P < 0.001 and ****P < 0.0001 by two-way ANOVA followed by the Sidak correction for multiple comparisons compared with the control. **J** **P < 0.01 by unpaired two-tailed Welch’s *t*-test compared with the control. All data are presented as the mean ± SEM values.

The intracellular NAD^+^ concentration is controlled by the balance between NAD^+^ consumption and production. Therefore, we next examined whether the rate of consumption and/or synthesis of NAD^+^ is altered depending on the strength of H_2_O_2_ stimulation. Since the NAD^+^ consumption in the early phase of H_2_O_2_ stimulation depends at least in part on PARP1 (Fig. 2A) which is activated by DNA damage, we compared the extent of DNA damage in response to 0.7 mM and 1.1 mM H_2_O_2_ stimulation by monitoring γH2AX. We found that the γH2AX intensity under 1.1 mM H_2_O_2_ was higher than that under 0.7 mM H_2_O_2_ at 6 and 10 h after stimulation (Fig. 3A), suggesting that 1.1 mM H_2_O_2_ stimulation induced stronger DNA damage than 0.7 mM stimulation. Then, we examined whether PARP1 activity is also increased in strong H_2_O_2_ stimulation by monitoring the accumulation of PARylated proteins (Fig. 3B). Under both 0.7 mM and 1.1 mM H_2_O_2_ stimulation, however, the observed accumulation of PARylated proteins was similar soon after stimulation (15 min) and decreased thereafter. The amount of PARylated protein was comparable between the 0.7 and 1.1 mM H_2_O_2_ stimulation conditions at all time points examined. The result suggested that the NAD^+^ consumption by PARP1 may not largely differ between 0.7 mM and 1.1 mM H_2_O_2_ stimulations.

We next focused on NAD^+^ production under oxidative stress. A major NAD^+^ synthesis system is the NAD^+^ salvage pathway, in which nicotinamide (NAM) phosphoribosyltransferase (NAMPT) is a rate-limiting enzyme (Fig. 3C) [34, 35]. Treatment with the NAMPT inhibitor FK866 almost completely suppressed the recovery of NAD^+^ upon 0.7 mM H_2_O_2_ stimulation (Fig. 3D), suggesting that NAD^+^ recovery relies on the NAMPT-dependent salvage pathway. Consistent with the NAD^+^ dynamics, FK866 treatment abolished ATP recovery under 0.7 mM H_2_O_2_ stimulation (Fig. 3E). Moreover, FK866 treatment enhanced LDH release and reciprocally suppressed caspase-3 activity even under 0.7 mM H_2_O_2_ stimulation (Fig. 3F, G), suggesting that FK866 treatment changed the form of cell death from apoptosis to necrosis by inhibiting NAD^+^ recovery under 0.7 mM H_2_O_2_ stimulation.

The NAD^+^ salvage pathway consists of two steps: (1) NAMPT-dependent nicotinamide mononucleotide (NMN) synthesis from NAM and phosphoribosyl diphosphate (PRPP) and (2) nicotinamide mononucleotide adenylyltransferase (NMNAT)-dependent NAD^+^ synthesis from NMN and ATP (Fig. 3C) [34, 35]. Administration of nicotinamide riboside (NR) increases the intracellular NAD^+^ concentration even under NAMPT-deficient conditions, because NMN can be synthesized from NR by NR kinase (NRK) (Fig. 3C) [36]. Thus, we treated cells with NR under 1.1 mM H_2_O_2_ stimulation to investigate whether NMNAT is functional even under strong oxidative stress. In cells treated with NR, NAD^+^ recovery was observed at 10 h after 1.1 mM H_2_O_2_ stimulation (Fig. 3H). NR treatment also rescued ATP recovery in the late phase (Fig. 3I), suppressed LDH release and increased caspase-3 activity in response to 1.1 mM H_2_O_2_ stimulation (Fig. 3J, K). These results indicate that NR treatment switches the form of cell death from necrosis to apoptosis via the recovery of intracellular NAD^+^ and ATP under strong oxidative stress. That is, although NMNAT is functional even under 1.1 mM H_2_O_2_ stimulation, a shortage of NMN caused by impairment of upstream system of the NAD^+^ salvage pathway leads to the failure of NAD^+^ recovery.

To investigate what upstream step is impaired in the NAD^+^ salvage pathway under 1.1 mM stimulation, we first examined the protein level of NAMPT by immunoblotting. The intracellular NAMPT level did not change by either weak or strong H_2_O_2_ stimulation (Fig. 3A). To examine NAMPT activity, we established a HeLa cell line that stably expressed Flag-tagged NAMPT. The cells were exposed to 0.7 or 1.1 mM H_2_O_2_ for 3 or 6 h, and Flag-NAMPT was immunoprecipitated. Then, the activity of purified NAMPT was measured by an in vitro assay. Unexpectedly, NAMPT activity did not change in response to stimulation with either concentration of H_2_O_2_ (Fig. 3L). Therefore, impairment of the NAD^+^ salvage pathway under strong oxidative stress is not attributable to a decrease in either the amount or activity of NAMPT, but instead to some other reasons, e.g., deficiency of NAMPT substrates, such as PRPP (Fig. 3C).

### Caspase-9 cleavage was impaired under strong oxidative stress

**Fig. 4:**
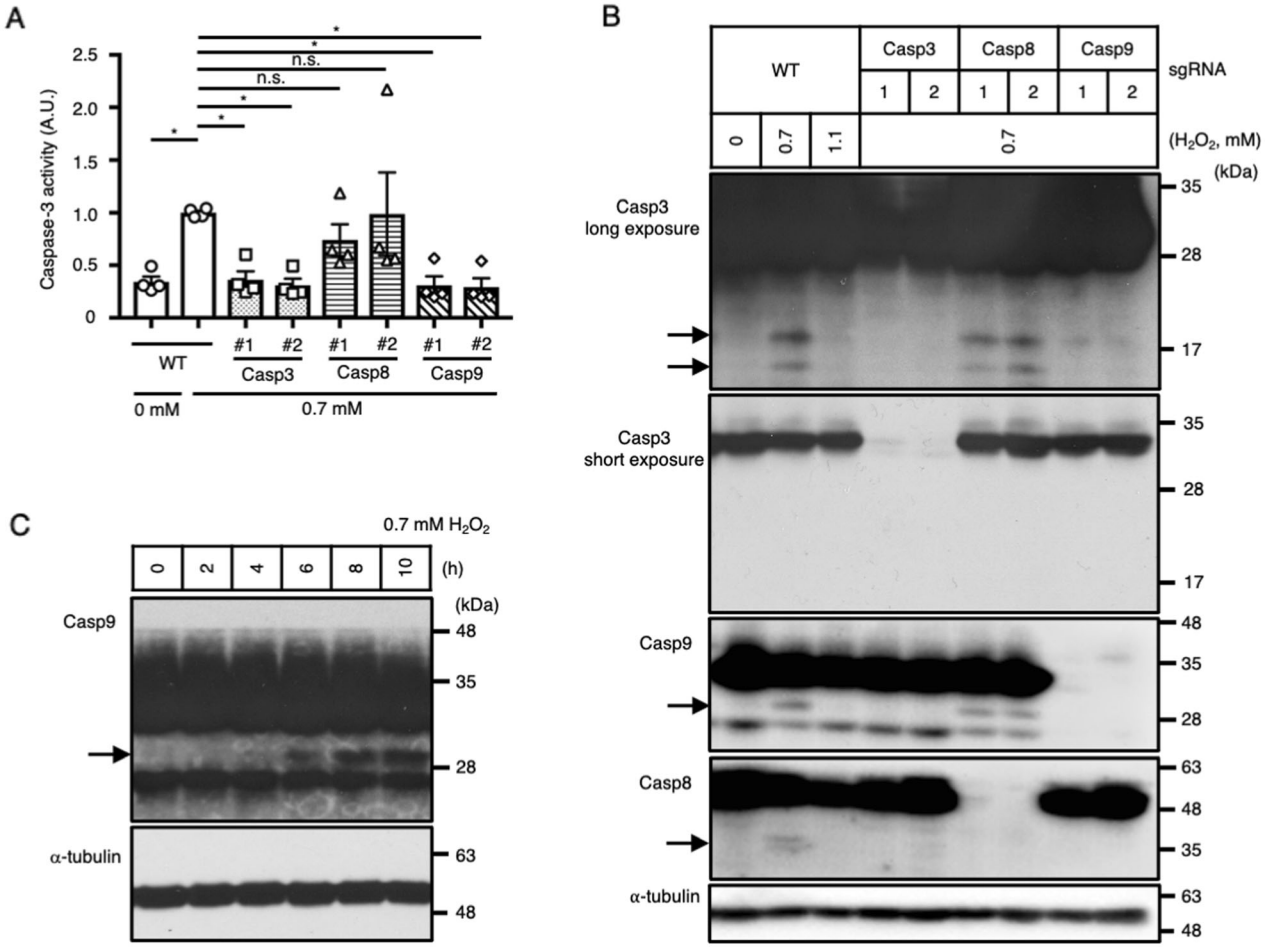
Caspase-9 cleavage was impaired under strong oxidative stress. **A** Caspase-3 activities in WT, caspase-3 KO (casp3), caspase-8 KO (casp8), and caspase-9 KO (casp9) HeLa cells stimulated with 0.7 mM H_2_O_2_ for 10 h (*n* = 4). **B** Cleavage of each caspase resulting from 0.7 or 1.1 mM H_2_O_2_ stimulation for 10 h, as detected by immunoblotting (*n* = 3). **C** Time course of caspase-9 cleavage resulting from 0.7 mM H_2_O_2_ stimulation, as detected by immunoblotting (*n* = 3). **A** *P < 0.05 by one-way ANOVA followed by Dunnett’s multiple comparisons test compared with WT cells with 0.7 mM H_2_O_2_ stimulation.

Finally, we sought to determine why ATP recovery correlates with apoptosis induction. Two major apoptotic pathways have been defined, namely, the extrinsic and intrinsic pathways [37]. The extrinsic pathway is induced through death receptor signaling and is dependent on caspase-8 as the initiator caspase. On the other hand, the intrinsic pathway is induced by various types of stress, including DNA damage and ER stress. In the intrinsic pathway, caspase-9 functions as the initiator caspase. To investigate which pathway is involved in the apoptosis induced by 0.7 mM H_2_O_2_ stimulation, we established caspase-8, -9 and -3 knockout HeLa cell lines by the CRISPR/Cas9 system. Caspase-3 activity was increased by 0.7 mM H_2_O_2_ stimulation in WT and caspase-8 knockout cells, whereas it was abolished in caspase-9 knockout cells (Fig. 4A), suggesting that apoptosis induction by 0.7 mM H_2_O_2_ stimulation is mediated by the caspase-9-dependent intrinsic pathway, as reported previously [38]. The intrinsic pathway consists of several molecular steps: (1) cyt c release from mitochondria; (2) apoptosome formation with cyt c, Apaf1 and caspase-9; (3) cleavage and activation of caspase-9 in the apoptosome; and (4) cleavage and activation of caspase-3 by activated caspase-9 [37]. Cleavage of each caspase under oxidative stress was monitored by immunoblotting (Fig. 4B). In WT HeLa cells, both caspase-3 and -9 were cleaved upon 0.7 mM H_2_O_2_ stimulation, but these cleavage events were impaired under 1.1 mM H_2_O_2_ stimulation, suggesting that the upstream step of caspase-9 cleavage is abrogated under strong oxidative stress. Cleavage of caspase-3 and -9 was detected in caspase-8 knockout HeLa cells, but caspase-3 cleavage was diminished in caspase-9 knockout cells under 0.7 mM H_2_O_2_ stimulation. Although caspase-3 is known to be a downstream caspase of caspase-9, caspase-3 knockout abolished caspase-9 cleavage, suggesting the existence of a positive feedback loop between caspase-3 and caspase-9 in 0.7 mM H_2_O_2_-induced apoptosis. Although caspase-8 cleavage was slightly detected in WT cells stimulated with 0.7 mM H_2_O_2_, caspase-8 cleavage had a negligible contribution to the induction of apoptosis, because caspase-8 knockout had no effect on caspase-3 activity (Fig. 4A) or cleavage of caspase-3 (Fig. 4B).

**Fig. 5:**
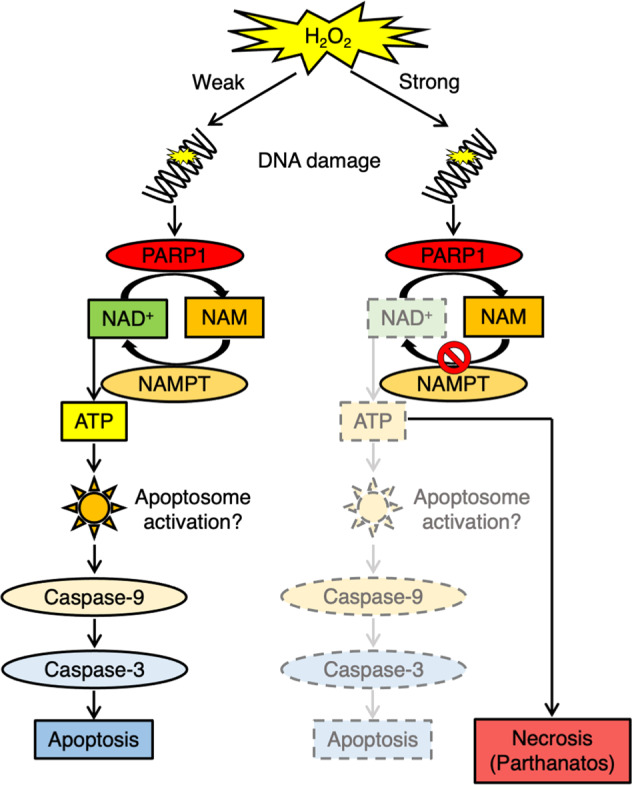
Schematic model of the switch between forms of cell death under H_2_O_2_ stimulation. Under weak H_2_O_2_ stimulation, NAD^+^ is consumed by PARP1 but resynthesized via the NAMPT-dependent salvage pathway. This NAD^+^ recovery is necessary for the synthesis of ATP and induction of the intrinsic apoptotic pathway. On the other hand, NAD^+^ resynthesis is abrogated upon strong H_2_O_2_ stimulation, probably because of the impairment of the NAMPT-dependent step in the NAD^+^ salvage pathway, resulting in the depletion of ATP, suppression of the intrinsic apoptotic pathway and induction of necrosis.

Events upstream of caspase-9 cleavage include cyt c release from mitochondria, apoptosome formation, and apoptosome activation. Among these events, apoptosome activation is known to require ATP or dATP [39]. Therefore, ATP recovery may be important for apoptosome activation. Consistent with this idea, cleavage of caspase-9 was detected after 6 h of 0.7 mM H_2_O_2_ stimulation (Fig. 4C), corresponding well to the time point when ATP recovery was observed (Fig. 1F). Collectively, these results suggest that strong H_2_O_2_ stimulation does not induce apoptosis because of the lack of ATP recovery after H_2_O_2_ stimulation, which impairs apoptosome activation and caspase-9 cleavage (Fig. 5).

## Discussion

In this study, we demonstrated that intracellular NAD^+^ and ATP dynamics regulated by the NAD^+^ salvage pathway were key factors in determining the form of cell death in H_2_O_2_-stimulated HeLa cells. Previous reports have shown that the preexisting intracellular ATP concentration alters the form of cell death induced by some stimuli, including Fas and hypoxia [16, 18]. In this report, we demonstrated that the dynamics of the intracellular ATP concentration during H_2_O_2_ stimulation differed between the apoptosis-inducing weak stimulation and the necrosis-inducing strong stimulation (Fig. 1F). In both weak and strong H_2_O_2_ stimuli, the ATP concentration decreased immediately after stimulation. However, the ATP concentration recovered in the late phase of weak but not strong H_2_O_2_ stimulation, suggesting that the ATP concentration in the late phase (after 6 h) but not in the early phase (after approximately 1 h) of stimulation determines the final form of cell death. The reason that the ATP concentration in the late phase determines the form of cell death may be attributed to the mechanism of apoptosis induction. We demonstrated that caspase-3 activation under weak oxidative stress required caspase-9 but not caspase-8, suggesting that the intrinsic pathway of apoptosis is responsible (Fig. 4A). Among the molecular mechanisms of the intrinsic pathway, activation of the apoptosome for caspase-9 cleavage has been reported to require ATP or dATP [39]. Consistent with this idea, caspase-9 cleavage was impaired under strong H_2_O_2_ stimulation, during which the intracellular ATP was completely depleted until 10 h after stimulation (Fig. 4B). Our speculation was further supported by the finding that cleavage of caspase-9 was observed only in the late phase (>6 h) of weak H_2_O_2_ stimulation when the intracellular ATP concentration had recovered (Fig. 4C).

We showed that the dynamics of NAD^+^ paralleled those of ATP under H_2_O_2_ stimulation (Fig. 2H). In HeLa cells, H_2_O_2_ stimulation decreased the intracellular NAD^+^ concentration in a PARP1 activity-dependent manner (Fig. 2A). Similar to the ATP concentration, the NAD^+^ concentration gradually recovered only under weak H_2_O_2_ stimulation (Fig. 2H). The result suggests that the strength of H_2_O_2_ stimulation affects NAD^+^ recovery, which eventually leads to the difference in the ATP concentration and thus the form of cell death. Consistent with this observation, inhibition of NAD^+^ depletion by a PARP1 inhibitor prevented ATP depletion (Fig. 2A, B) and changed the form of cell death from necrosis to apoptosis under strong H_2_O_2_ stimulation (Fig. 2C, D). Previous reports showed that DNA-damaging stimuli induce necrotic cell death in a PARP1-dependent manner, a phenomenon called parthanatos [26]. In this study, we exhibited a tight correlation between the switch of the form of cell death and NAD^+^ dynamics in H_2_O_2_-stimulated HeLa cells. Whether this NAD^+^ dynamics-dependent switching system can be applied to other parthanatos-inducing stimuli is an interesting question. Since parthanatos is known to associate with various diseases, such as neurodegenerative diseases and ischemia-reperfusion injury [26], it is important to examine the generality of cell death form switching by the intracellular NAD^+^ and ATP concentrations in other context of parthanatos.

Regarding the difference of NAD^+^ dynamics under weak and strong H_2_O_2_ stimulation, there are two factors that affect the intracellular NAD^+^ concentration: the rate of consumption and the rate of synthesis. A major NAD^+^-consuming enzyme in the early phase of H_2_O_2_ stimulation was PARP1, because the PARP1 inhibitor substantially suppressed the NAD^+^ depletion in the early phase of H_2_O_2_ stimulation (Fig. 2A). Therefore, difference in PARP1 activity could affect the intracellular NAD^+^ dynamics. However, the PARP1 activity showed no apparent difference between weak and strong H_2_O_2_ stimulation at any time points (Fig. 3B), suggesting that the speed of NAD^+^ consumption by PARP1 may not be largely affected by the concentrations of H_2_O_2_ used in this study. These data implied that the rate of NAD^+^ synthesis rather than the rate of NAD^+^ consumption may be different depending on the strength of H_2_O_2_ stimulation in the late phase. In this study, we found that NAD^+^ recovery under weak H_2_O_2_ stimulation was mediated by the NAMPT-dependent NAD^+^ salvage pathway (Fig. 3D). In addition, we found that NR treatment, which can increase the NAD^+^ concentration in an NMNAT-dependent but NAMPT-independent manner, recovered the NAD^+^ concentration 10 h after strong H_2_O_2_ stimulation (Fig. 3H). These data suggested that the NMNAT-dependent step is intact but upstream step, such as NAMPT-dependent step, is suppressed under strong H_2_O_2_ stimulation. Under NR treatment, recovery of intracellular ATP began earlier than that of NAD^+^ (Fig. 3H, I). The result seems contradictory to the idea that NAD^+^ is required to produce ATP. However, although the difference was not statistically significant, the concentration of intracellular NAD^+^ in NR-treated samples was slightly higher than that in control samples after 6 h of 1.1 mM H_2_O_2_ stimulation (Fig. 3H). Since NAD^+^ functions as a coenzyme for ATP generation, NAD^+^ itself is not consumed during ATP generation. Therefore, it is possible that even a small amount of NAD^+^ might be sufficient to recover the ATP concentration under strong H_2_O_2_ stimulation.

Because the NAMPT-dependent step was a plausible determinant whether to recover NAD^+^ under H_2_O_2_ stimulation, we compared the NAMPT protein amount and activity in H_2_O_2_-stimulated HeLa cells. However, neither the amount of intracellular NAMPT protein nor the activity of purified NAMPT changed upon stimulation with any concentration of H_2_O_2_ (Fig. 3A, L), implying that the amounts of other component(s), such as NAMPT substrates, may be different depending on the strength of H_2_O_2_ stimulation. Further analysis of the NAMPT-dependent step is needed to elucidate the reasons that the dynamics of the intracellular NAD^+^ concentration differ depending on the strength of H_2_O_2_ stimulation; this knowledge will clarify a new molecular mechanism by which cells perceive the strength of H_2_O_2_ stimulation.

Oxidative stress-induced cell death is reported to be involved in many kinds of disorders, including neurodegenerative diseases, ischemia-reperfusion injury, liver disorders and cancer [12, 40, 41]. Since these diseases are affected by the inflammatory milieu, which is exacerbated by necrotic cell death, the proper control of the form of cell death based on our findings in the present study (manipulation of NAD^+^ recovery) would provide a treatment strategy for these diseases. In support of this idea, previous studies showed that an increase in the intracellular ATP concentration prevented progression of neurodegenerative diseases in a mouse model [42–46]. In this study, using HeLa cells, we demonstrated the threshold of switching the form of cell death fell around 1 mM H_2_O_2_, which is relatively high for physiological conditions [47]. It is considerable that other non-tumor cells, like primary neurons might show lower switching threshold, according to resistance to oxidative stress. Further analysis is important to investigate the variety of the switching threshold depending on cells, comparing with their capacity of the NAD^+^ salvage pathway. Collectively, our findings indicate the importance of a NAD^+^ recovery system to determine the form of cell death under oxidative stress and provide a new target for manipulating the NAD^+^ and ATP concentration as a potential strategy for the treatment of oxidative stress-related disorders.

## Materials and methods

### Cell lines and cell culture

HeLa cells and HeLa cells with KO of each caspase were cultured in DMEM-low glucose (Sigma, Cat#D6046) supplemented with 10% fetal bovine serum (FBS). HeLa cells stably expressing Flag-NAMPT or GFP were cultured in DMEM-low glucose supplemented with 10% FBS and 1 μg/mL puromycin (Thermo Fisher Scientific, Cat#A11138-03). HEK293T cells were cultured in DMEM-high glucose (Sigma, Cat#D5796) supplemented with 10% FBS. All cells were cultured in 5% CO_2_ at 37 °C and verified to be negative for mycoplasma contamination.

Caspase-3, -8 and -9 knockout HeLa cells were generated using the CRISPR/Cas9 system. To create the KO vectors, we used the following sets of DNA oligonucleotides.

### Caspase-3 #1

CACCGTACCCGGGTTAACCGAAAGG and AAACCCTTTCGGTTAACCCGGGTAC

### Caspase-3 #2

CACCGTCTTACCCGGGTTAACCGAA and AAACTTCGGTTAACCCGGGTAAGAC

### Caspase-8 #1

CACCGAACGAGATATATCCCGGATG and AAACCATCCGGGATATATCTCGTTC

### Caspase-8 #2

CACCGTCCGGGATATATCTCGTTTG and AAACCAAACGAGATATATCCCGGAC

### Caspase-9 #1

CACCGCCGCCGATCCGCTTCGTCCA and AAACTGGACGAAGCGGATCGGCGGC

### Caspase-9 #2

CACCGACAATCTTCTCGACCGACAC and AAACGTGTCGGTCGAGAAGATTGTC

Each set of DNA oligonucleotides was annealed in TE buffer by incubation at 95 °C for 5 min and subsequently at 60 °C for 5 min. Annealed DNA was then inserted into PX459 (Addgene, Cat#62988) digested with BbsI.

HeLa cells were transfected with PX459 containing separate sgRNAs targeting each caspase with Lipofectamine 2000 (Thermo Fisher Scientific, Cat#11668019) according to the manufacturer’s protocols with minor optimization. To reduce cytotoxicity, the cell culture medium was replaced with fresh medium 6 h after transfection. Two days after transfection, the medium was changed to puromycin-containing medium (1 μg/mL) and incubated for two more days. Then, polyclonal KO cells were seeded onto 10 cm dishes at a low density without puromycin for picking of single clones. Single clones of each caspase KO cell line were confirmed by immunoblotting.

To establish HeLa cells with stable expression of Flag-NAMPT or GFP, each cDNA was cloned into a pLenti CMV/TO Puro DEST (670-1) (Addgene, Cat#17293) plasmid, and lentiviral transduction was performed. Briefly, to produce lentivirus, HEK293T cells were transfected with the constructed plasmids, pCMV-VSV-G (Addgene, Cat#8454), and psPAX2 (Addgene, Cat#12260) using Lipofectamine 3000 (Thermo Fisher Scientific, Cat#L3000015), according to the manufacturer’s protocols with minor optimization. To reduce cytotoxicity, the cell culture medium was replaced with fresh medium 4 h after transfection. Lentivirus-containing culture supernatants were collected at 48 h post transfection and filtered through a 0.45 μm pore size filter (Millipore, Cat#SLHV033RS). HeLa cells were seeded into 24-well plates (5 × 10^4^ cells/well) in medium supplemented with 10 μg/mL polybrene (Nacalai Tesque, Cat#17736-44), and the cells were transduced with lentiviral vectors overnight. The next day, the culture medium was replaced with fresh medium containing puromycin (1 μg/mL) for selection of transduced cells.

### Antibodies

For the immunoblotting experiment, we used the following antibodies: rabbit monoclonal anti-PARP1 antibody (EPR18461: abcam, Cat#ab191217, 1/5000), mouse monoclonal anti-Actin antibody (AC-40: Sigma, Cat#A3853, 1/10000), rat monoclonal anti-α-tubulin antibody (YL1/2: Santa Cruz Biotechnology, Cat#sc-53029, 1/20000), rabbit polyclonal anti-PAR antibody (Enzo Life Science, ALX-210-890A-0100, 1/5000), rabbit polyclonal anti-NAMPT antibody (BETHYL, Cat#A300-372A, 1/10000), rabbit polyclonal anti-caspase-3 antibody (Cell Signaling, Cat#9662, 1/2000), mouse monoclonal anti-caspase-8 antibody (1C12: Cell Signaling, Cat#9746, 1/2000), mouse monoclonal anti-caspase-9 antibody (C9: Cell Signaling, Cat#9508, 1/2000), rabbit polyclonal anti-AIF antibody (Cell Signaling, Cat#4642, 1/5000), mouse monoclonal anti-Histone H2AX antibody (322105: R&D SYSTEMS, Cat#MAB3406, 1/1000) and rabbit polyclonal anti-γH2AX antibody (abcam, Cat#ab2893, 1/1000). We also used a horse anti-mouse IgG, HRP-linked antibody (Cell Signaling, Cat#7076); a goat anti-rabbit IgG, HRP-linked antibody (Cell Signaling, Cat#7074); and a goat anti-rat IgG, HRP-linked antibody (Cell Signaling, Cat#7077).

### H_2_O_2_ stimulation

Two days before stimulation, cells were seeded in 6-, 24-, or 96-well culture plates (2 × 10^5^, 5 × 10^4^ or 1 × 10^4^ cells/well, respectively). 1.5 h prior to stimulation, the culture medium was replaced with 2 mL, 500 μL or 100 μL of fresh medium, respectively. DPQ (Cayman, Cat#14450, 50 μM), FK866 (Sigma, Cat#F8557, 10 nM), NR (Carbosynth, Cat#NN15702, 1 mM) or vehicle control (DMSO ( ≥ 99.5% purity: Sigma, Cat#D5879) or H_2_O) was administered at the same time. DPQ and FK866 were diluted with DMSO to 50 mM and 10 μM, respectively. NR was diluted with H_2_O to 1 M. H_2_O_2_ (FUJIFILM Wako, Cat# 081-04215) was diluted to 100× the final concentration with H_2_O, and the diluted H_2_O_2_ was added to the culture medium at 20, 5 or 1 μL/well for 6-, 24-, or 96-well culture plates, respectively.

### LDH assay

Necrosis induction was measured using an LDH Cytotoxicity Assay (FUJIFILM Wako, Cat#299-50601). Culture medium was collected 30 h after H_2_O_2_ stimulation and centrifuged for 3 min at 400 × g (medium sample). Cells were lysed with PBS containing 0.1% Triton X-100, and the cell lysate was then centrifuged for 10 min at 17,700 × g (lysate sample). The medium and lysate samples were individually mixed with reagents in 96-well microplates, and the absorbance was measured at 570 nm using a Varioskan Flash (Thermo Fisher Scientific) after incubation for approximately 5 min at room temperature. Necrosis induction was evaluated by calculating LDH release (%) as follows: (absorbance (abs) of medium samples − background)/((abs of lysate samples − background) + (abs of medium samples − background)).

### Caspase-3 activity assay

Apoptosis induction was measured using a fluorogenic substrate for activated caspase-3, Ac-DEVD-AFC (Cayman, Cat#14459). Cells were lysed with PBS containing 0.1 % Triton X-100 10 h after H_2_O_2_ stimulation, and the cell lysate was centrifuged for 10 min at 17,700 × g. Lysate samples were individually mixed with reagents in 384-well microplates (15 μL of lysate sample, 25 μL of 2 × Reaction Buffer (Bio Vision, Cat#1068), 7.25 μL of PBS, 2.5 μL of caspase-3 substrate (1 mM in DMSO) and 0.25 μL of 1 M dithiothreitol (TCI, Cat#D1071)). Fluorescence signals were measured at specific wavelengths (Ex/Em = 400/505 nm) using a Varioskan Flash after incubation for approximately 90 min at 37 °C. For normalization, the protein amount in each lysate sample was measured using a DC^TM^ protein assay (Bio-Rad, Cat#5000113, #5000114, #5000115). Caspase-3 activity in each sample was calculated as follows: ((fluorescence intensity of sample − background)/protein concentration of sample (μg/μL)). In Figs. 2D, 3G, K, caspase-3 activity in each sample was normalized to caspase-3 activity induced by 0.7 mM H_2_O_2_ without any treatment under the same experimental conditions (data not shown in the figure). In Fig. 2G, caspase-3 activity induced by 0.7 mM H_2_O_2_ without any knockdown treatment was used for normalization (data not shown in the figure). Finally, we defined the arbitrary unit (A.U.) by dividing each caspase activity value by the average caspase-3 activity in the technical replicates of the control sample under stimulation with 0.7 mM H_2_O_2_.

### Annexin V staining

Apoptotic cells were stained with Annexin V-FITC (Nacalai Tesque, Cat#15342). Twenty-four hours after H_2_O_2_ or STS (FUJIFILM Wako, Cat#197-10251) stimulation, the medium was changed to new medium containing 50 μL of Annexin V-FITC (1/200) and Hoechst 33342 (DOJINDO, Cat#H342, 1/1000), and the cells were incubated for 30 min in 5% CO_2_ at 37 °C. The ratio of Annexin V-FITC-positive cells was calculated with the CellInsight NXT platform (Thermo Fisher Scientific).

### Gene silencing by siRNA transfection

Knockdown experiments with siRNA were carried out by reverse transfection using Lipofectamine RNAiMAX (Thermo Fisher Scientific, Cat#13778150) and Opti-MEM (Thermo Fisher Scientific, Cat#31985) according to the manufacturer’s protocol. The final concentrations of PARP1 and AIF siRNA were 0.2 nM and 0.25 nM, respectively. The PARP1 siRNAs were purchased from Dharmacon (siGENOME Human PARP1 (142) siRNA #1 (D-006656-03-0010), target sequence: GCAACAAACUGGAACAGAU and siGENOME Human PARP1 (142) siRNA #2 (D-006656-04-0010), target sequence: GAAGUCAUCGAUAUCUUUA), and the AIF siRNAs were purchased from Thermo Fisher Scientific (Stealth siRNA Human AIF #1, target sequence: GGGUUAAGGUGAUGCCCAAUGCUAU and Stealth siRNA Human AIF #2, target sequence: GGAGUCAGCAGUGGCAAGUUACUUA). siGENOME Nontargeting siRNA #1 (Dharmacon) and Stealth siRNA Negative Control Medium GC Duplex #1 (Thermo Fisher Scientific) were used as controls. Each experiment was performed 2 days after transfection.

### Cell lysis and immunoblotting

In Figs. 2E, 4B, C and S1A, cells seeded in 24-well plates were lysed with 100 μL/well lysis buffer (10 mM EDTA (pH 8.0), 150 mM NaCl, 20 mM Tris–HCl (pH 7.5), 1% sodium deoxycholate, 1% Triton X-100, 1 mM phenylmethylsulfonyl fluoride, and 5 µg/mL leupeptin). Cell extracts were centrifuged for 10 min at 17,700 × g, and supernatants were prepared by adding 2 × SDS sample buffer (80 µg/mL bromophenol blue, 10 mM dithiothreitol, 28.8% glycerol, 4% SDS and 80 mM Tris-HCl (pH 8.8)) and boiling at 98 °C for 3 min.

In Fig. 3A, B, cells seeded in 24-well plates were lysed with 100 μL/well 2 × SDS sample buffer heated to 98 °C. Cell extracts were boiled at 98 °C for 3 min and sonicated with an Ultra Sonic Homogenizer UH-50 (SMT).

Proteins were resolved by SDS-PAGE and electroblotted onto Immobilon-P membranes (Millipore, Cat#IPVH00010). The membranes were blocked with 2.5% skim milk (Megmilk Snow Brand) in TBS-T (137 mM NaCl, 20 mM Tris-HCl (pH 8.0) and 0.1% Tween 20) and probed with the appropriate primary antibodies diluted in primary antibody dilution buffer (TBS-T supplemented with 5% BSA (Iwai Chemicals, Cat#A001) and 0.1% NaN_3_ (Nacalai Tesque, Cat#312-33)) overnight. After replacing the primary antibody solution and probing the membranes with the appropriate HRP-conjugated secondary antibodies diluted with 2.5% skim milk in TBS-T, antibody-antigen complexes were detected on X-ray films (FUJIFILM, Cat#47410-26615 or Cat#47410-22617) or with a FUSION Solo S imaging system (VILBER) using ECL reagents (GE Healthcare). Representative data are shown in the figures, and more than two additional experimental replicates showed similar results. The images of uncropped gels are shown in Supplementary Fig. 2. Quantification was performed via densitometry using Fusion Capt software (VILBER).

### ATP assay

The intracellular ATP concentration was measured using “Cell” ATP Assay reagent Ver. 2 (FUJIFILM Wako, Cat#381-09306). After H_2_O_2_ stimulation in a 96-well white plate (Corning, Cat#353377), the medium was removed, and 50 μL of ATP reaction mixture (1:1 medium: assay reagent) was added. Samples were incubated at room temperature in the dark for 10 min, and luminescence was measured using a Varioskan Flash. The ATP amount in each sample was calculated as follows: (luminescence of each sample–background). The relative ATP concentrations in Figs. 2B, 3E, I were calculated by comparison to the ATP amount at 1.5 h after the medium change (that is, 0 h of stimulation without any treatment). Finally, we defined the A.U. by dividing each ATP concentration by the average ATP concentration in the technical replicates of the control sample at 0 h.

### NAD^±^ assay

The intracellular NAD^+^ concentration was measured using an Amplite Fluorimetric Total NAD and NADH Assay Kit (AAT Bioquest, Cat#15257). After H_2_O_2_ stimulation, cells were lysed with PBS containing 0.1% Triton X-100 and 1 mM nicotinamide (TCI, N0078), and the cell lysate was then centrifuged for 10 min at 17,700 × *g*. Lysate samples were individually mixed with reagents in 384-well microplates (15 μL of lysate sample and 15 μL of NAD/NADH working solution), and fluorescence was measured at specific wavelengths (Ex/Em = 540/590 nm) using a Varioskan Flash approximately 30 min after incubation at room temperature. For normalization, the protein amount in each lysate sample was measured using a DC^TM^ protein assay. The NAD^+^ amount in each sample was calculated as follows: ((fluorescence intensity of sample–background)/protein concentration of sample (μg/μL)). The relative NAD^+^ concentrations in Figs. 2A, 3D, H were calculated by comparison to the NAD^+^ amount at 1.5 h after the medium change (0 h of stimulation without any treatment). Finally, we defined the A.U. by dividing each NAD^+^ concentration by the average NAD^+^ concentration in the technical replicates of the control sample at 0 h.

### NAMPT assay

NAMPT activity was measured using a CycLex NAMPT Colorimetric Assay Kit Ver. 2 (Cat#CY-1251V2). HeLa cells stably expressing Flag-NAMPT or GFP were seeded into 6-well plates. Two days after seeding, cells were stimulated with H_2_O_2_ and lysed with 500 μL/well lysis buffer (20 mM Tris (pH 8.0), 250 mM NaCl, 1 mM EDTA, 1 mM EGTA, 1% Triton X-100, 1 mM phenylmethylsulfonyl fluoride, and 5 µg/mL leupeptin). Cell extracts were centrifuged for 10 min at 17,700 × *g*, and the protein concentrations in the supernatants were measured using a DC^TM^ protein assay. A 450 μL aliquot from the sample with the lowest protein concentration was transferred to a new tube. Other samples were also transferred to new tubes according to each protein concentration to equalize the total protein amount to that in the sample with the lowest concentration. The samples were diluted to 450 μL with lysis buffer. The diluted samples were incubated with anti-FLAG antibody beads (FUJIFILM Wako, clone 1E6, Cat#016-22784) for 1 h at 4 °C. The beads were washed with lysis buffer three times before use, and 20 μL of the bead suspension (50% slurry in lysis buffer) was used for immunoprecipitation. After incubation, the beads were washed twice with lysis buffer and twice with PBS, followed by direct addition of the reaction mix (total 90 μL: 5 μL of #1, 3.6 μL of #2 to #9, 56.2 μL of H_2_O; #1 to #9 are reagents provided in the NAMPT assay kit). Then, the samples were incubated at 1200 rpm and 30 °C using a ThermoMixer C (Eppendorf). At 30, 60, 90 and 120 min after the start of incubation, 20 μL of the reaction mix supernatants was aliquoted into 384-well microplates, and the absorbance was measured at 450 nm using a Varioskan Flash. Reaction mix without the supernatant was used as the sample at 0 min of incubation. After incubation, the remaining beads were prepared by adding 50 μL of 2 × SDS sample buffer and boiling at 98 °C for 3 min. The amount of NAMPT in the samples was quantified by immunoblotting as described above. NAMPT activity in each sample was calculated as follows: ((abs of each sample at 120 min–abs at 0 min)/band density of NAMPT in the corresponding sample determined by immunoblotting). Relative NAMPT activity was calculated by comparison to the NAMPT activity at 1.5 h after the medium change (0 h of stimulation). Finally, we defined the A.U. of NAMPT activity by dividing each NAMPT activity value by the average NAMPT activity in the technical replicates of the sample stimulated with 0 mM H_2_O_2_.

### Statistical analysis

All data are presented as the mean ± SEM values. The numbers of samples, sample sizes, and statistical tests are indicated in the figure legends. The investigators were not blinded to the group allocation during the experiment. Statistical tests were performed using GraphPad Prism 7.0c or excel, and *P* < 0.05 was considered statistically significant. All experiments that are subjected to statistical analysis have more than three samples in each condition. Unpaired two-tailed Student’s *t*-test, unpaired two-tailed Welch’s *t*-test, one-way ANOVA followed by Dunnett’s multiple comparisons test, one-way ANOVA followed by the Tukey-Kramer multiple comparisons test, two-way ANOVA followed by the Sidak correction for multiple comparisons, or two-way ANOVA followed by Dunnett’s multiple comparisons test was used in this study. F-test was performed before *t*-test to estimate the variances between group of data. Throughout: *P < 0.05, **P < 0.01, ***P < 0.001 and ****P < 0.0001.

## Supplementary information

Figure S1

Figure S2

## Data Availability

All data generated or analyzed during this study are included in this published article and its supplementary information files.
