# Supplementary figures and images for: NAMPT-dependent NAD+ salvage is crucial for the decision between apoptotic and necrotic cell death under oxidative stress
Source: Cell Death Discov. 2022 Apr 11;8:195. doi: 10.1038/s41420-022-01007-3 (PMC9001718; doi:10.1038/s41420-022-01007-3)

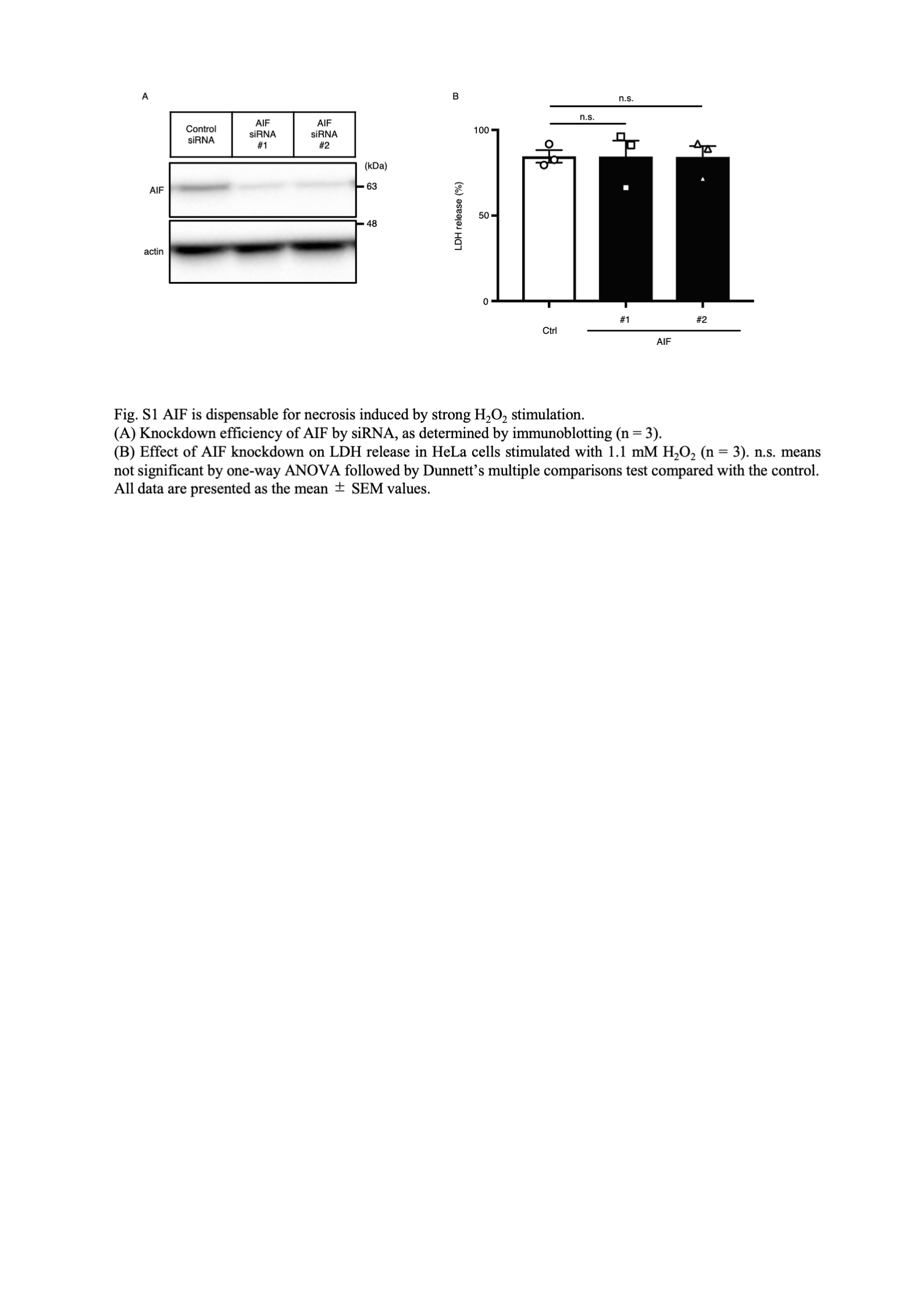

Supplement: Supplementary file 1 — Figure S1 [file 41420_2022_1007_MOESM1_ESM.png]

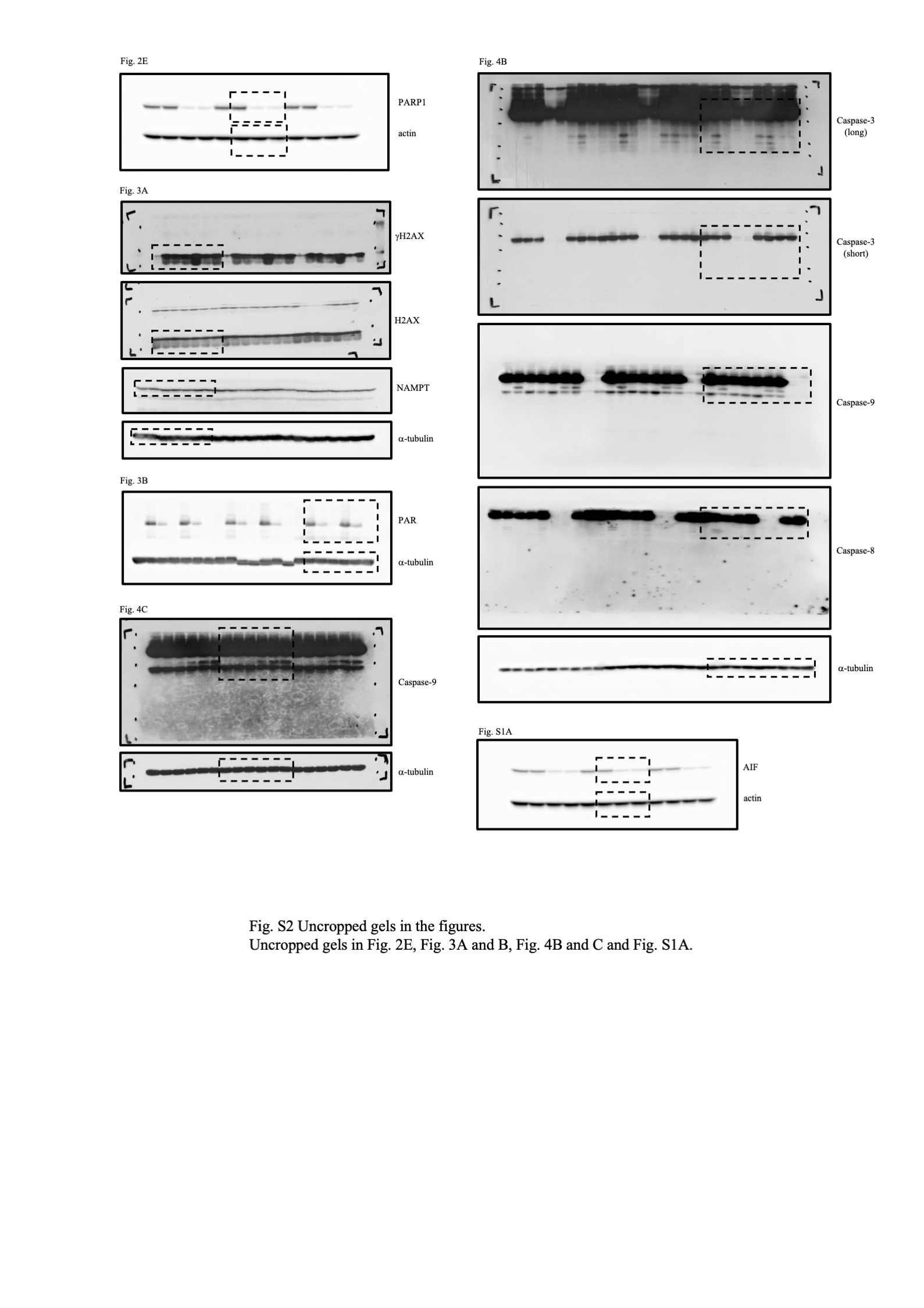

Supplement: Supplementary file 2 — Figure S2 [file 41420_2022_1007_MOESM2_ESM.png]
